# Photothermal Therapy for the Treatment of Glioblastoma: Potential and Preclinical Challenges

**DOI:** 10.3389/fonc.2020.610356

**Published:** 2021-01-15

**Authors:** Chiara Bastiancich, Anabela Da Silva, Marie-Anne Estève

**Affiliations:** ^1^Aix-Marseille Univ, CNRS, INP, Inst Neurophysiopathol, Marseille, France; ^2^Aix Marseille Univ, CNRS, Centrale Marseille, Institut Fresnel, Marseille, France; ^3^APHM, Hôpital de la Timone, Service Pharmacie, Marseille, France

**Keywords:** glioblastoma, photothermal therapy, nanoparticles, hyperthermia, preclinical studies

## Abstract

Glioblastoma (GBM) is a very aggressive primary malignant brain tumor and finding effective therapies is a pharmaceutical challenge and an unmet medical need. Photothermal therapy may be a promising strategy for the treatment of GBM, as it allows the destruction of the tumor using heat as a non-chemical treatment for disease bypassing the GBM heterogeneity limitations, conventional drug resistance mechanisms and side effects on peripheral healthy tissues. However, its development is hampered by the distinctive features of this tumor. Photoabsorbing agents such as nanoparticles need to reach the tumor site at therapeutic concentrations, crossing the blood-brain barrier upon systemic administration. Subsequently, a near infrared light irradiating the head must cross multiple barriers to reach the tumor site without causing any local damage. Its power intensity needs to be within the safety limit and its penetration depth should be sufficient to induce deep and localized hyperthermia and achieve tumor destruction. To properly monitor the therapy, imaging techniques that can accurately measure the increase in temperature within the brain must be used. In this review, we report and discuss recent advances in nanoparticle-mediated plasmonic photothermal therapy for GBM treatment and discuss the preclinical challenges commonly faced by researchers to develop and test such systems.

## Introduction

Glioblastoma (GBM) is a very aggressive primary malignant brain tumor and finding effective therapies is a pharmaceutical challenge and an unmet medical need. Despite great advances in molecular understanding, identification of predictive factors and technological advances for patient’s treatment and care, only a few drugs are currently approved for GBM and we are still far from reaching a cure (1, 2). The standard of care therapy for GBM consists in surgical resection of the tumor, followed by radiotherapy and adjuvant plus concomitant chemotherapy with Temozolomide (TMZ) (Stupp protocol) (3). Since 2015, a medical device based on tumor-treating (TT)-Fields can also be applied on supratentorial GBM patients. However, the median overall survival of GBM patients following this aggressive therapeutic regimen remains less than 2 years (20.9 months TTFields and TMZ; 16 months TMZ) (4).

**Figure 1:**
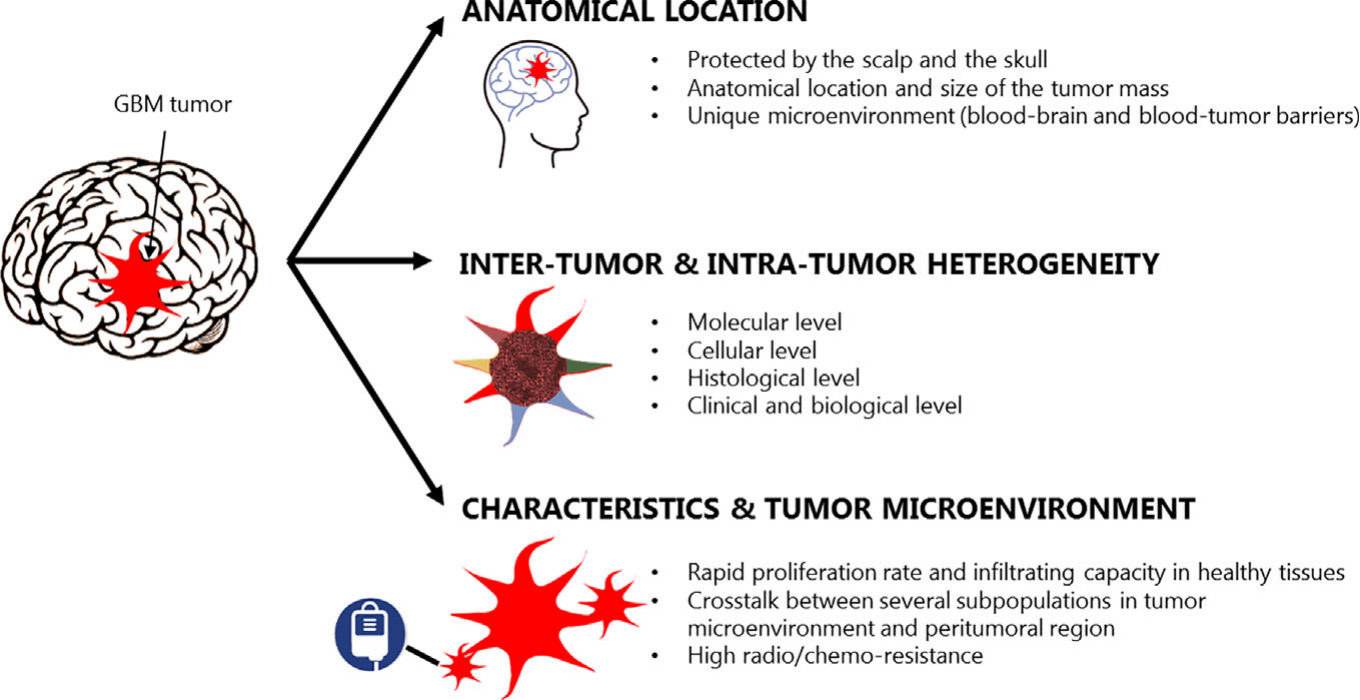
Glioblastoma characteristics and therapeutic challenges, adapted from (5).

From a clinical perspective, the effective treatment of GBM is limited by several challenges (**Figure 1**). Firstly, the anatomical location and size of the tumor impact the extent of resection, as a fine balance must be achieved between the maximal removal of malignant tissue and minimal operative risk (6). When safe and gross cytoreduction is achieved, residual infiltrating GBM cells undetectable by advanced imaging techniques still remain in the peritumoral area leading to disease progression and inevitable patient’s death (7, 8). Secondly, the central nervous system (CNS) has a unique microenvironment and is protected by the blood-brain barrier (BBB), which limits the access of systemically delivered drugs to the brain reducing the therapeutic options available for GBM. Additionally, GBM is characterized by high inter-tumor and intra-tumor heterogeneity at a cellular, molecular, histological, and clinical level leading to very different responses to therapeutic agents and failure of targeted therapies (9, 10). This leads to very poor and unchanged prognosis despite drug delivery advances. Finally, GBM presents rapid proliferation rate and infiltrating capacity in the healthy brain tissue thanks to the co-existence and crosstalk of several subpopulations within its microenvironment. For example, a subset of astrocytoma cells with ultra-long, thin, and highly dynamic membrane protrusions named tumor microtubes are able to extend in the surrounding tissue for tumor cell invasion, proliferation, and connection between tumor cells (11). At the same time, a subpopulation of highly tumorigenic glioma stem cells (GSCs) with high plasticity and self-renewal properties contribute to tumor malignancy through their sustained proliferation, invasion, stimulation of angiogenesis, suppression of anti-tumor immune responses and chemo-resistance (12). Moreover, many other non-cancerous cells (e.g. reactive astrocytes, tumor-associated macrophages and microglia, etc.) residing within the tumor or in the peritumoral area can regulate cancer progression and treatment responses characterizing the unique cellular microenvironment of GBM (13). The constant search for effective long-term treatments for GBM has prompted investigations in a number of directions, including hyperthermia (HT) (14, 15).

HT is based on the local application of high temperatures in body tissues to induce irreversible cell injury in the targeted region and is considered a promising therapeutic approach for cancer (16, 17). Temperature increase during a certain time induces cell membrane collapse, protein denaturation, arrest in enzyme function, and mitochondrial dysfunction leading to tumor apoptosis and coagulative necrosis (16). The advantage of HT as cancer treatment is based on the fact that cancer tissues are more thermosensitive than normal tissues—probably due to their acidic interstitial environment, reduced heat dissipation capacity, and increased metabolic stress—allowing maximum anti-tumor effectiveness with limited damage to the surrounding healthy tissues (18). When the tip of a heating applicator is directed towards the tumor site, it generates a temperature gradient and a lesion characterized by three different areas: 1) a central zone with maximal temperature increase (≥50°C), where irreversible damage is induced; a peripheral zone characterized by sublethal HT (41–45°C), where cells undergo apoptosis or recover following injury; a surrounding zone which remains unaffected by the ablation (16). Several thermal ablative modalities exist depending on the energy source and its delivery mode (interstitial or non-invasive), but their common challenge for the treatment of non-superficial tumors is to achieve even heat distribution and therapeutic temperatures at the tumor site (17, 19). The use of laser-induced HT, photodynamic therapy (PDT), microwave HT, high-intensity focused ultrasound (HIFU)-induced HT, magnetic HT, and radiofrequency HT in brain tumors has already been reviewed elsewhere (17, 20–22). In this review, we will summarize the main advances in nanoparticle (NP)-mediated plasmonic photothermal therapy (PTT) for GBM treatment and discuss the preclinical challenges commonly faced by researchers to develop and test such systems.

## Potential and Preclinical Challenges of Nanoparticles-Mediated Photothermal Therapy for Glioblastoma

PTT is a non-invasive therapy that requires the use of an external near-infrared (NIR) laser to irradiate the tumor either topically or interstitially (through an optical fiber), and a photoabsorbing (PTA) agent able to accumulate at the tumor site. Upon laser radiation, the PTA agent harvests the light energy, converts and releases it as heat, inducing localized HT that leads to partial or complete tumor ablation (19, 23). NP with intrinsic optical properties (*e.g.* Au-based or other semiconducting metal-based NPs, magnetic Fe_3_O_4_ NPs, carbon-based nanomaterials) or loaded with small PTA molecules (*e.g.* cyanine or porphyrin derivatives) are ideal PTA agents for PTT (24). As the energy conversion and heat dissipation only occurs when the frequency of the incident light is overlapped with the absorption band of the PTA agent, this must present strong NIR absorbance coefficient. Indeed, the NIR region corresponds to the transparent window for biological tissues and light in this range of wavelengths (approx. 650–900 nm) can penetrate through soft tissues with minimum absorption, avoiding loss of energy prior to irradiation of the PTA agent (23). Moreover, NPs for PTT should be non-toxic in absence of laser exposure and have physicochemical properties (*e.g.* shape, size, zeta potential, stability, etc.) that allow them to be administered systemically and accumulate efficiently in the tumor *via* passive or active targeting.

**Figure 2:**
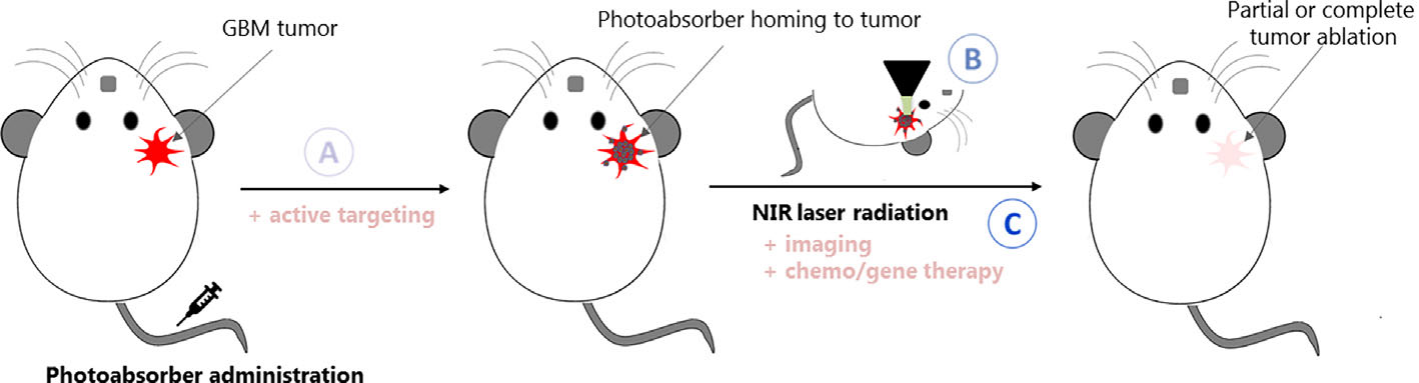
Schematic representation of the challenges to face while developing PTT systems for GBM: **(A)** the PTA agent needs to cross the BBB to reach the tumor site; **(B)** *in situ* (and possibly in depth) thermometry must be put in place to monitor the temperature increase within the brain; **(C)** the laser and PTA agent parameters must be carefully set and adequate to induce localized HT at the tumor site, which might be deep inside the brain. Strategies such as active targeting, multi-modal imaging or chemo/gene therapy can be coupled to increase the therapeutic effect of GBM PTT.

PTT may be a promising strategy for the treatment of GBM, as it allows the destruction of the tumor using heat as a non-chemical treatment for disease bypassing the GBM heterogeneity limitations, conventional drug resistance mechanisms and side effects on peripheral healthy tissues. However, its development is hampered by the distinctive features of this tumor as summarized in **Figure 2**. PTA NPs need to reach the tumor site at therapeutic concentrations, bypassing the BBB upon systemic administration. Subsequently, a NIR light irradiating the head must cross multiple barriers (scalp, skull, and healthy brain tissue) to reach the tumor site without causing any local damage. Its power density needs to be within the safety limit [maximum permissible exposure: 1.0 W/cm^2^ and 0.33 W/cm^2^ for 1,064 and 808 nm lasers, respectively (25)] and its penetration depth should be sufficient to induce deep and localized HT and achieve tumor destruction. To properly monitor the therapy, imaging techniques that can accurately measure the increase in temperature within the brain must be used.

Several authors have described the use of innovative NPs for GBM PTT in preclinical studies but, to our knowledge, no clinical study has reported yet their use in glioblastoma patients. Some authors have only evaluated their systems *in vitro* (**Table 1**), while others have achieved to demonstrate an efficacy *in vivo* overcoming some of the intrinsic challenges of GBM PTT (**Tables 2** and **3**). In the tables, the parameters that we consider more relevant are highlighted in order to show the differences and similarities between the studies. Unfortunately, important parameters (*e.g.* mass extinction coefficient, laser beam size, effective delivered laser dose, NP concentration, long-term survival studies) are missing in some of the reported studies. Moreover, the lack of standardized protocols to evaluate the NPs PTT efficacy (*e.g.* treatment incubation time, tumor size at treatment) makes it hard to properly compare the reported nanomaterials. Therefore, we have limited our analysis to a precise description of the advances in the field emphasizing those studies that are, in our opinion, better conceived and more promising.

**Table 1:** Non-exhaustive list of *in vitro* cellular studies reporting NPs for GBM PTT.

Photoabsorbing agent		Size range	Laser conditions			Glioma cell line	Model	Reference
			λ	Power (W/cm^2^)	Exposure time (min)			
**Metal based NP**	AuNR	41 × 10 nm	808 nm	1.2 W*	20	1321N1 human astrocytoma	2D	(26)
	Nes-AuNR	28 × 9 nm	800 nm	0.5	7 or 4	X01 GBM, X01 GBM-BMP	2D, 3D	(27)
				2.5**	4		3D	
	AuNS	SiO_2_ core: 100 nm Au shell: 10 nm	800 nm	80	2	U373, U87 MG	2D	(28)
	Ma-AuNS	SiO_2_ core: 120 nm Au shell: 15 nm	810 nm	2, 7, 14, or 28	5 or 10	ACBT human glioma	2D, 3D	(29, 30)
	Ma-AuNR	45 × 15 nm						
	AuNSt@probe	35 nm	808 nm	2	5	U87 MG	2D	(31)
	AuNSt-ICG-BSA	80 nm	808 nm	1	5	U87 MG	2D	(32)
	CPT-GNC	73 nm	790 nm	76**	0.75	42 MG-BA	2D	(33)
	Ir_1_-AuSiO_2_ NP	57 nm	254 nm	4	60	U87 MG	2D	(34)
	TiN NP	4–40 nm	670 nm	4.4	10	U87 MG	2D, 3D	(35)
**Carbon-based NP**	nano-rGO-RGD	Lateral: 20 nm	808 nm	15.3	8	U87 MG	2D	(36)
	nanoGO-Tf-FITC	20–200 nm	808 nm	7.5	5	U251 glioma	2D	(37)
	PVP-G	70–360 nm	808 nm	2	3	U251 glioma	2D	(38)
	DOX-GMS-PI	Lateral: 50−150 nm	808 nm	6	5	U251 glioma	2D	(39)
	IUdR-PLGA-NGO	83 nm	808 nm	2	3	U87 MG	2D	(40)
	MWCNTS	150–190 nm	970 nm	3	0–1.5	U87 MG, U373, D54	2D, 3D	(41)
	PDA-ICG-NDs	358 nm	808 nm	2 W*	5	U-118 MG	2D	(42)
**Others**	ICG-PL-PEG	18 nm	808 nm	0.75 to 3.25	5	U87 MG	2D	(43)
	FA-Au-NP	55 nm	808 nm	0.8	10	C6 glioma	2D	(44)

*power density; **pulsed laser average power density; AuNR, Au nanorods; Nes, nestin; PEG, polyethylene glycol; AuNS, Au@SiO_2_ nanoshells; Ma, macrophages; AuNSt, Au nanostars; AuNSt@probe, AuNSt conjugated with Atto 655 dye, Asp-Glu-Val-Asp peptide and a folic acid; ICG, indocyanine green; BSA, bovine serum albumin; CPT, camptothecin; GNC, mesoporous silica coated Au nanocluters; NP, nanoparticles; AuSiO_2_, silica NPs with a gold core; TiN, titanium nitride; nano-rGO, nanosized reduced graphene oxide; RGD, arginine–glycine–aspartic acid peptide; Tf, transferrin; FITC, fluorescein isothiocyanate; PVP-G, polyinylpyrrolidone-coated graphene sheets; GMS-PI, interleukin 13 peptide modified mesoporous silica-coated graphene nanosheet; DOX, doxorubicin; IUdR, 5-iodo-2-deoxyuridine; PLGA, poly lactic-coglycolic acid; NGO, nanographene oxide functionalized with poly lactic-coglycolic acid; MWCNTS, phospholipid-poly(ethylene glycol) coated multiwalled carbon nanotubes; PDA, polydopamine; NDs, nanodiamonds; PL-PEG, phospholipid-polyethylene glycol; FA, folic acid; Au-NP, Au decorated polymeric NP.

**Table 2:** Non-exhaustive list of *in vivo* preclinical PTT efficacy studies performed using subcutaneous GBM models.

Photoabsorbing agent		Functions(other than PTT)	PTT laser and treatment conditions				Preclinical model	Reference
			Power (W/cm^2^)	Exposure time (min)	Tumor size at treatment	Administration route		
**Metal based NP**	RVG29-SiO_2_-PEG-AuNR	AT	1.5	5	200–300 mm^3^	iv	N2a neuroblastoma	(45)
	^64^Cu-RGD-Au NR	AT; CT	1	10	100 mm^3^	iv	U87 MG	(46)
	AuNS	–	4	3	∼3–5 mm	iv	U373 GBM	(47)
	RGD-AuNSt	AT; PAI	1	10	∼8–12 mm	iv, multiple	U87 MG	(48)
	PPDI-PEG-Au NP	PAI	0.3	5	70 mm^3^	iv	U87 MG	(49)
**Carbon-based NP**	rGONM-PEG-Cy7-RGD	AT; FI	0.1	7	50 mm ^3^	iv	U87 MG	(50)
	PNG-RGD	AT; FI	2.5	5	∼6 mm	it	U87 MG	(51)
	C225-EPI-PEG-NGO	AT; FI; chemo	2	2	N/A	iv	U87 MG	(52)
**Hybrid NPs or others**	rGO-AuNRVe-DOX	PAI; chemo	0.25	5	60 mm^3^	iv	U87 MG	(53)
	pDNA-loaded AuNR-Fe_3_O_4_NS	PAI; CT; MRI; gene therapy	2	5	150 mm^3^	it, multiple	C6	(54)
	C225-Au-MNP	AT; MFH	0.3	30	∼5 mm	pt, multiple	U251	(55)
	^125^I-RGD-PEG-MNP	AT; CT; MRI	0.5	5	200 mm^3^	iv, multiple	U87 MG	(56)
	ANG-Au-PLGA-DTX NPs	AT; CT; chemo	1.5	1.5	100 mm^3^	iv, multiple	U87 MG	(57)
	UCNP-PEG-ICG-TOS-RGD	AT; CT; UCLI; chemo	0.5	5	∼4–6 mm	iv, multiple	U87 MG	(58)
	ASQ-DOX-PGEA2/p53 nanohybrids	PAI; CT; FI; gene therapy; chemo	2	5	100–200 mm^3^	it, multiple	C6	(59, 60)
	^125^I RGD-CR780-PEG NPs	AT; CT; FI; PAI	0.5	10	100 mm^3^	iv	U87 MG	(61)
	melittin/ICG peptide nanofiber hydrogel	FI; PAI; chemo	2	8	75 mm^3^	it	C6	(62)
	CuS–Fn NCs	CT; PAI	0.8	5	60 mm^3^	iv	U87 MG	(63)
	PPyHMs	USI	0.64	10	300 mm^3^	it	U87 MG	(64)
	holo-Tf-ICG	FI; PAI	0.8	5	60 mm^3^	iv	U87 MG	(65)
	CPNP	PAI	0.8	5	80 mm^3^	iv	U87 MG	(66)

All the studies were performed using 808 nm continuous-wave NIR laser except: 635 nm (55); 790 nm (48); 1064 nm (67) lasers.

AT, active targeting; chemo, chemotherapy; CT, X-ray computed tomography; FI, fluorescence imaging; MFH, magnetic fluid hyperthermia; MRI, magnetic resonance imaging; PAI, photoacoustic imaging; PTT, photothermal therapy; UCLI, upconversion luminescence imaging; USI, ultrasound imaging; it, intratumoral; iv, intravenous; pt, peritumoral; NP, nanoparticles; RVG29, 29-residue peptide rabies virus glycoprotein; PEG, polyethylene glycol; AuNR, gold nanorods; RGD, arginine–glycine–aspartic acid peptide; AuNS, Au@SiO_2_ nanoshells; AuNSt, gold nanostars; PPDI, poly(perylene diimide); rGO, reduced graphene oxide; rGONM, rGO nanomesh; Cy7, cyanine 7; PNG, porphyrin immobilized nanographene oxide; C225, cetuximab; EPI, epirubicin; rGO-AuNRVe, hybrid reduced graphene oxide-loaded ultrasmall gold nanorod plasmonic vesicles; DOX, doxorubicin; pDNA, plasmid DNA; Fe_3_O_4_NS, Fe_3_O_4_ nanospheres; Au-MNP, core-shell Fe_3_O_4_@Au magnetic nanoparticles; MNP, Fe@Fe_3_O_4_ magnetic nanoparticles; ANG, angiopep-2 peptide; PLGA, poly (lactide-co-glycolide); DTX, docetaxel; UCNP, cesium-based upconversion nanoparticles; ICG, indocyanine green; TOS, alpha-tocopheryl succinate; ASQ-PGEA2, multifunctional hetero-nanoparticles comprising Au NRs, mesoporous silica, quantum dots and two-armed ethanolamine-modified poly(glycidyl methacrylate) with cyclodextrin cores; CP NP, donor-acceptor conjugated polymer nanoparticles; CR780, croconaine; CuS–Fn NCs, ultrasmall copper sulfide NPs loaded inside the cavity of ferritin nanocages; PPyHMs, polypyrrole hollow microspheres; holo-Tf, holo-transferrin nano assemblies.

**Table 3:** Non-exhaustive list of *in vivo* preclinical PTT efficacy studies performed using orthotopic GBM models.

Photoabsorbing agent	Functions(other than PTT)	PTT laser and treatment conditions				Preclinical model	Notes	Reference
		Power (W/cm^2^)	Exposure time (min)	Administration regimen	I/I time			
Ma-AuNS	–	N/A	10	it	N/A	C6	• Optical laser fiber into the brain	(68)
cRGD-PEG-HAuNS	AT; PAI	16	3	iv	24 h	U87 MG-Luc	• MRTI maps, T_max_: 58°C	(69)
VEGF-AuNS	AT	3	6	iv	24 h	U373 GBM	• Cranial glass window • Intravital microscopy	(70)
Tf-TPGD	AT; chemo	2.5	5	iv, multiple	24 h	C6	• Thermal texture map system, at 12h T_max_: 38°C	(71)
HCCD	FI; PAI	1	5	iv, multiple	30 min	U87 MG		(72)
OMCN–PEG–Pep22/doxycycline	AT; chemo	N/A	5	iv, multiple	N/A	C6	• Skull bone removal • Thermal imager, T_max_: 55°C	(73)
ANG-IMNPs	AT; PDT	0.21	3	iv	8 h	ALTS1C1 astrocytoma		(74)
cRGD-CPNP	AT; PAI	0.8	5	iv	24 h	U87 MG-Luc	• Thermal camera, T_max_: 48°C	(67)
BLIPO-ICG	AT; FI	1	5	iv	12 h	C6-Luc	• Scalp removal • IR thermal imaging, T_max_: 48°C	(75)

All the reported studies were performed using 808 nm continuous-wave NIR laser except: 810 nm (68); 1,064 nm (67) lasers.

N/A, not applicable; I/I time, time between treatment injection and laser irradiation; AT, active targeting; chemo, chemotherapy; FI, fluorescence imaging; IR, infrared; MRTI, magnetic resonance temperature imaging; PAI, photoacoustic imaging; PDT, photodynamic therapy; PTT, photothermal therapy; it, intratumoral; iv, intravenous; AuNS, Au@SiO_2_ nanoshells; VEGF, vascular endothelial growth factor; Ma-NS, AuNS loaded macrophages; cRGD, cyclic arginine–glycine–aspartic acid peptide; PEG, polyethylene glycol; HAuNS, hollow gold nanospheres; Tf, transferrin; TPGD, nanoscale graphene oxide loades with doxorubicin; HCCD, Highly crystalline carbon nanodots; OMCN, oxidized nanocrystalline mesoporous carbon particles; Pep22, Pep22 polypeptide; ANG, angiopep-2 peptide; IMNP, hybrid nano-assemblies loaded with IR-780 (PTT agent) and mTHPC (PTD agent); CP NP, donor-acceptor conjugated polymer nanoparticles; BLIPO-ICG, biomimetic proteolipid nanoparticles; ICG, indocyanine green.

## *In Vitro* Cellular Studies Concerning Nanoparticles-Mediated Photothermal Therapy for Glioblastoma

*In vitro* cellular studies are essential to demonstrate the biocompatibility of NPs, test the PTT potential, imaging properties, or anticancer efficacy of a PTA agent. They are also essential to evaluate the mechanisms of cell death on which the PTT effect relies, to assess and compare different PTA agents prior to preclinical *in vivo* studies and to set the therapeutic parameters (*e.g.* NPs concentration, laser power, time of exposure). Several *in vitro* GBM models exist (primary cells *vs* immortalized cell lines, monolayer cell cultures *vs* spheroids, etc.) and their complementary use can be cost effective while providing a wide range of information: recently, the advantages of using 3D cell cultures compared to 2D models to test the efficacy of NP-based PTT have been highlighted by Darrigues et al. (76).

### Metal-Based Nanomaterials

Fernandez Cabada et al. evaluated the ability of gold nanorods (AuNR) to act as heat-inducing agents in 1321N1 GBM cells (26). The PTT cytotoxic effect induced by laser irradiation in presence of AuNR was extensively evaluated showing increased cell death compared to the controls when cells were exposed to the correct concentration of AuNR. The authors investigated further the mechanisms involved in the cell death mediated by AuNR following laser exposure, showing that tumor cells exposed to treatment were necrotic (and not apoptotic) and PTT damage causes a significant loss of cell membrane integrity. As the cell membrane is the most susceptible cellular component to thermal damage and only a small percentage of AuNR were efficiently internalized in GBM cells (where they accumulated in subcellular organelles), the authors suggest that cancer cell death may have occurred by extracellular HT. Gonçalves et al. developed AuNR functionalized with nestin (Nes) for the active targeting of infiltrating GSCs, which are one of the main responsible for GBM chemo-resistance and the onset of recurrences (27). Nes-AuNRs were selectively internalized in by GSCs *via* energy-dependent and caveolae-mediated endocytosis and induced cells apoptosis following NIR irradiation. Their cytotoxic activity on the X01 GBM and X01 GBM-BMP cell lines (expressing 99 or 29% Nes^+^ cells, respectively) was more sensitive to PTT treatment in 2D cell monolayers compared to 3D tissue-mimicking models (800 nm laser, 7 min, 0.5 mW/cm^2^). These 3D models were established by culturing cells in matrixmetalloproteinase-responsive multi-arm poly(ethylene glycol) (starPEG)/heparin hydrogels at different stiffness, to recreate the normal brain and GBM tissue microenvironments. The two cell lines were also co-cultured in the 3D model showing that X01 GBM cells were more strongly affected by the laser treatment compared to X01 GBM-BMP cells (73 *vs* 29% dead cells, respectively). However, this experiment was performed using different laser parameters compared to the previous one (800 nm pulsed laser, 100 fs pulse length, 80 MHz repetition rate; 2.5 mW/cm^2^ average power density, 4 min). Despite the promising results reported in this work using sophisticated 3D models and the accurate evaluation of therapy resistance behavior in 2D *vs* 3D cell models, no *in vivo* studies were performed to confirm the potential Nes-AuNRs targeting GSCs as GBM treatment.

Bernardi et al. used PEGylated interleukin-13 receptor-alpha 2 antibody-tagged gold-silica nanoshells (PEG-IL13Rα2-AuNS) as PTT strategy against U373 and U87 MG glioma cells (28). Nanoshells (NS) are optically tunable NPs formed by a spherical dielectric core surrounded by a thin metal shell: depending on the relative core and shell thickness, NS can either absorb or scatter the light at a desired wavelength and convert the absorbed NIR into heat (28, 29). IL13Rα2-AuNS were composed of a 100 nm silica core surrounded by a 10 nm Au shell and showed a peak light absorption at 800 nm. Once internalized selectively by tumor cells overexpressing IL13Rα2 and upon exposure to a continuous wave (CW) laser (800 nm; 80 W/cm^2^, 2 min), they generated enough heat to kill them, minimizing the damage in the surrounding healthy cells. Baek et al. used murine macrophages (P388D1 cells; Ma) as delivery vehicles for AuNS for GBM PTT (29). Ma can efficiently uptake NS which aggregates in vacuoles dispersed throughout their cytoplasm and have a natural ability to traverse the intact and compromised BBB (77). *In vitro*, Ma-AuNS were able to infiltrate into human grade IV GBM (ACBT cells) spheres, where they acted as PTA agents. Indeed, laser irradiation of the Ma-AuNS/glioma cells spheroids (810 nm; laser power 7, 14, or 28 W/cm^2^; 5 or 10 min) showed significant growth inhibition compared to controls. Cell death following PTT was observed only in Ma-AuNS and tumor cells adjacent to them, while spheroids containing empty Ma were not affected by the laser exposure confirming the efficacy of the Ma-AuNS as PTA agents. The same group also compared Ma-AuNS and Ma-AuNR, showing greater PTT efficacy of AuNS in multicellular hybrid spheroids formed by loaded Ma (either Ma-AuNS or Ma-AuNR) and ACBT cells upon laser exposure (810 nm; laser power 2, 7, 14, or 28 W/cm^2^; 10 min) (30). The better efficacy of AuNS is explained by their larger cross-sectional area which confers them increased absorption and conversion efficiency of NIR light to heat compared to AuNR, and theoretical calculations suggested that the AuNR to AuNS ratio required to obtain equivalent PTT effect is 10:1 (78). This group also evaluated the PTT efficacy of AuNS loaded in rat alveolar macrophages (NR8383) in C6 cells *in vitro* (monolayers and spheroids) and *in vivo* models (68). In an orthotopic C6-rat model, intratumoral (it) administration of AuNS was followed by the insertion of an optical fiber to irradiate the tumor area (810 nm, 1 W, 10 min) and thus avoid the scalp and skull. Even though the authors obtained good PTT antitumor efficacy of Ma-AuNR (confirmed by tumor growth suppression 13 days post-treatment), they failed to proof the BBB-crossing properties of this system as they administered Ma-AuNS intratumorally. Even though it was shown that exogenously loaded Ma are unable to traverse the BBB in healthy rat brain (79), it would be interesting to evaluate if the BBB disruption observed in GBM-bearing animals is enough to achieve sufficient Ma-AuNR tumor uptake for PTT.

Wang et al. developed a theranostic agent for simultaneous tumor targeting PTT and fluorescent turn-on self-therapeutic imaging (AuNSt@probe) (31). This system was formed of a NIR fluorescent dye (Atto 655), a caspase-3 responsive peptide linker (Asp-Glu-Val-Asp), a folic acid (FA) targeting moiety, and a PTT transducer (gold nanostars; AuNSt). U87 MG monolayers were incubated with AuNS@probe and exposed to laser irradiation (808 nm; 2 W/cm^2^; 5 min): cells death was induced by the caspase-3-dependent apoptosis. This induced the cleavage of the peptide-linker which turned on the fluorescence dye, allowing an imaging-guided feedback for accurate apoptosis and self-therapeutic effect monitoring. Chen et al. developed a bovine serum albumin stabilized gold nanostar-indocyanine green (AuNSt -ICG-BSA) nanoprobe to precisely monitor temperature variations in real time during PTT by surface enhanced Raman scattering (SERS) imaging (32). This actively targeted multifunctional nanoplatform showed excellent properties as SERS imaging probe and intracellular thermometer with a spatial resolution of subcellular level and a sensitivity of 0.37°C. Moreover, it showed good biocompatibility, cell uptake, and PTT performance following laser irradiation (808 nm; 1 W/cm^2^; 5 min) on U87 MG cells demonstrating high potential as theranostic probe.

Small Au nanoclusters protected by a mesoporous silica scaffold containing the cytotoxic drug camptothecin (CPT) were developed by Botella et al. (33). These systems showed good cytotoxic effect in 42-MG-BA human glioma cells following femtosecond pulsed laser irradiation (790 nm, 130 ± 6 fs pulse width, 76 MHz repetition rate, 600 mW; 76 W/cm^2^, 45 s). Cell death was achieved by the combination of the local thermal heating effect corresponding to absorption of the NIR radiation, the mechanical disruption of cellular components due to *in situ* generation of vapor microbubbles and the cytotoxic effect induced by intracellular drug release. Ricciardi et al. developed gold core-silica shell NPs incorporating a water soluble bioluminescent iridium(III) compound (Ir_1_-AuSiO_2_) as theranostic agents for simultaneous cellular imaging, PTT and PDT (34). Ir_1_ was selected as photosensitizer and luminescent probe thanks to its high emission quantum yield both in presence and absence of molecular oxygen, and its emission band centered at 515 nm. Ir_1_-AuSiO_2_ were spherical and highly monodispersed NPs with a size smaller than 100 nm. Their efficacy as PTT was tested in U87 MG cells, where concentrations <0.5 µM induced significant cancer cell death.

As an alternative to Au, titanium nitride (TiN) was also used as PTA agent as it is commercially available, thermoresistant and inexpensive (80). TiN NP with simple geometries can be easily synthetized, show localized surface plasmon resonance band within the biological transparency window, and high photothermal conversion efficiency (80). He et al. have shown the potential for PTT and photoacoustic (PA) imaging for cancer treatment *in vitro* and on a subcutaneous (sc) preclinical model of breast cancer following it or systemic administration of TiN NP (81). Our group has recently demonstrated *in vitro* the potential of TiN NPs as PTA agent for GBM PTT (35). In our study, 4–40 nm spherical TiN NP with a plasmonic peak around 640–700 nm were synthetized by femtosecond laser ablation and fragmentation in water or organic (acetone) solutions. *In vitro* tests demonstrated good internalization of TiN NP *via* endocytosis and an acceptable toxicity profile on human microvascular endothelial cells (HMEC–1) and U87 MG cells. Indeed, less than 30% of reduction in cell viability was observed after exposure at concentrations up to 10 mg/L after 3 days (monolayers) or 13 days (spheroids) confirming the low cytotoxicity profile of a TiN NPs in 2D and 3D U87 MG glioma cell culture models. Finally, U87 MG spheroids treated with TiN NP (25 nm, 10 mg/L in water) for 4 days and then exposed during 10 min to a CW laser beam (670 nm, 4.4 W/cm²) showed a significant decrease in both in size and viability, while the laser alone had no anti-proliferative effect. These results confirm the strong photothermal effect using TiN NP as sensitizers of IR radiation-induced HT, demonstrating the potential of laser-synthesized TiN NP as a PTA agent for GBM (35).

### Carbon-Based Nanomaterials

A wide range of carbon-based nanomaterials have been described for PTT because they present relatively high absorbance in the biological window, good biocompatibility, easy-to-modify surfaces, and good dispersibility in biological fluids (82). Robinson et al. reported the use of single-layered non-covalently PEGylated reduced graphene oxide (rGO) nanosheets for GBM PTT (36). Nano-rGO sheets (20 nm in average lateral dimension) were grafted with the arginine–glycine–aspartic acid (RGD) peptide and showed good biocompatibility, high NIR light absorbance, selective cellular uptake in U87 MG glioma cells, and active photoablation *in vitro* (808 nm, 15.3 W/cm², 8 min). Moreover, nano-rGO sheets can be loaded non-covalently with doxorubicin (DOX) and could therefore be used to synergistically combine photothermal effect and chemotherapy. Li et al. synthetized transferrin (Tf) and fluorescein isothiocyanate (FITC) functionalized nano-GO particles for targeted fluorescence imaging and GBM PTT (37). Tf-mediated transcytosis could increase nano-GO-Tf-FITC BBB crossing and tumor cells recognition and endocytosis. Nano-GO-Tf-FITC exhibited good biocompatibility and low cytotoxicity. Once internalized in U251 cells, they allowed for fluorescent imaging and successfully killed tumor cells upon laser exposure (808 nm, 7.5 W/cm², 5 min).

Markovic et al. compared the PTT anticancer activity of polyvinylpyrrolidone-coated graphene sheets (PVP-G) and single-wall carbon nanotubes (CNT) in U251 glioma cells (38). Thanks to their better dispersivity, PVP-G showed superior photothermal sensitivity and induced apoptotic and necrotic death *in vitro via* caspase activation/DNA fragmentation and cell membrane damage, respectively. Wang et al. used mesoporous silica-coated graphene nanosheet (GMS) as bifunctional vector for chemo-photothermal therapy in GBM (39). Their particles were grafted with an interleukin-13 (IL-13) residue peptide (named PI) for active targeting and loaded with chemotherapeutic drug DOX (GMS-PI-DOX). The PTT heating effect of GMS-PI is GMS-concentration and laser power intensity-dependent. This system presents pH-sensitive and heat-stimulative drug release, which could greatly enhance the therapeutic effect based on the targeting accumulation of GMS-PI-DOX in the tumor. The synergistic effect of GMS-PI-DOX chemotherapy and PTT was demonstrated *in vitro* in U251 glioma cells (808 nm, 6 W/cm^2^, 5 min). The authors also optimized their nanocomposite using magnetic graphene in the synthesis process (leading to MGMS-PI-DOX), to integrate magnetic targeting and MRI contrast agent capacity to the previously mentioned properties (active targeting, chemotherapy, PTT) (83). High MGMS-PI-DOX tumor uptake was observed in a U251 orthotopic *in vivo* model thanks to the combination of IP and magnetic-mediated targeting 2h-post intravenous (iv) administration and magnetic targeting. Unfortunately, no *in vivo* PTT antitumor efficacy studies were reported for this multifunctional theranostic platform.

Kargar et al. used nanographene oxide functionalized with poly lactic-coglycolic acid as a carrier of 5-iodo-2-deoxyuridine (IUdR-PLGA-NGO) for combined radiotherapy and PTT (40). Heating-dependent sustained release of IUdR was observed *in vitro*, which could be importantly accelerated by laser exposure. *In vitro* cellular studies on U87 MG cells demonstrated good cytotoxic activity (reduced colony forming ability) upon treatment with IUdR-PLGA-NGO followed by X-ray (2 Gy) plus laser radiation (808 nm, 2 W/cm^2^, 3 min).

Eldridge et al. developed multiwalled carbon nanotubes (MWCNTS) coated with phospholipid-PEG (PL-PEG) to optimize their stability in physiologic solution and treatment diffusion at the tumor site while maintaining the ability to achieve ablative temperatures upon laser exposure (41). MWCNTS showed high diffusion through brain extracellular matrix-mimicking phantoms following convection enhanced delivery (CED) infusion (4–5 mm from the infusion site in all directions). Interestingly, and rightfully enough, the authors demonstrated that MWCNTS do not induce a heat shock response (HSR) in GBM cell lines (U87 MG, U373, D54). This analysis was important as inherent or acquired thermotolerance (*e.g.* induced by sub-lethal heat conditioning) can limit the efficacy of thermal ablative therapies. Finally, *in vitro* studies performed on GBM cell lines (U87 MG, U373, D54 cells) but also on multicellular tumor spheroids showed efficacy of carbon nanotube mediated thermal therapy at modest concentrations (20 μg/ml) and laser power/duration (970 nm, 3 W/cm^2^, 90s).

Maziukiewicz et al. conjugated nanodiamonds with biomimetic polydopamine and indocyanine green (PDA-ICG-NDs) for PTT in GBM cells (42). This system was synthetized and characterized, and its efficacy as PTA agent was demonstrated in U-118 MG cells monolayers by WST-1 cell proliferation assay and flow cytometry following irradiation (808 nm, 2W, 5 min).

### Other Nanomaterials

Zheng et al. developed a nanoprobe by noncovalent self-assembly of PL-PEG and indocyanine green (ICG) (43). Two targeting moieties, αvβ3 monoclonal antibody (mAb) and FA, were also conjugated to the surface of the ICG-PL-PEG to evaluate its targeting properties on different cancer models. The targeted nanoprobe showed high uptake *via* ligand-receptor mediated endocytosis into U87 MG cells overexpressing integrin αvβ3, and induced tumor cell lethality upon treatment and laser exposure (808 nm, 5 min) in a laser power-dependent manner (55–35% viable cells at 1.25 and 1.75 W/cm^2^; 25% viable cells from 2.25 W/cm^2^).

Finally, Keyvan Rad et al. developed FA-conjugated gold-decorated polymer NPs (PGPNP) to achieve enhanced C6 glioma cells uptake, photo-generation of reactive oxygen species (ROS) upon UV irradiation and PTT following NIR laser exposure (808 nm, 0.8 W/cm^2^, 10 min) *in vitro* (44).

## *In Vivo* Preclinical Studies Concerning Nanoparticles-Mediated Photothermal Therapy for Glioblastoma

The *in vivo* validation step is essential prior to any clinical evaluation in the cancer patient, and the choice of the most appropriate model depends on the scientific objective pursued (*e.g.* description of a biological phenomenon, predictive evaluation of the behavior, efficacy and/or safety of new diagnostic or therapeutic system) (84). Several rodent models are available for GBM but GBM cells xenografts are the most used to test PTA NPs. Indeed, they can recapitulate some of the key GBM features, present high engraftment and growth rates but are also highly reproducible and reliable models. Transplantation models can be syngeneic if they involve the grafting of murine GBM cells into immune-competent animals of the same species and strain (*e.g.* C6 rat cells grafted into Wistar rats) or xenogeneic if human GBM cells are grafted in immunodeficient animals (*e.g.* U87 MG cells in nude mice). Grafts can be established by sc injection of GBM cells to be easily visualized and measured (heterotopic transplantation), or by intracranial injection of GBM cells to recapitulate the brain-specific tumor microenvironment and be more clinically relevant (orthotopic transplantation) (85).

### Metal-Based Nanomaterials

Viral-inspired NPs mimicking the virus in size, shape, and surface properties can increase NP cellular uptake and systemic residence leading to improved therapeutic efficacy (45). For example, a short peptide derived from the rabies virus glycoprotein (RVG29) specifically interacts with the nicotinic acetylcholine receptor (nAChR) expressed in neuronal cells (*e.g.* N2a cells), enabling the transvascular delivery of the virus to the brain. This peptide has previously shown to be useful for brain delivery of several small interfering RNA (86) and RVG-grafted nanocarriers (87). By synthesizing AuNRs with *i)* a shape closest to that of rabies-virus, *ii)* a silica-shell coating mimicking the virus matrix protein, and *iii)* by binding on their surface RVG29 at a density closer to the one on rabies-virus, Lee et al. obtained viral-mimetic RVG29-SiO_2_-PEG-AuNR able to bypass the BBB while preserving their localized surface plasmon resonance for PTT (45). Following iv administration of the carrier in N2a tumor-bearing mice, RVG29-SiO_2_-PEG-AuNR efficiently reached the tumor site and showed high temperature increase (>50°C in sc tumors; photothermal camera) following laser exposure (808 nm, 1.5 W/cm^2^, 5 min). Moreover, PTT tumor growth suppression was demonstrated both in sc and orthotopic models in the previously mentioned conditions. These very promising results were obtained in a brain neuroblastoma model, and it would be interesting to evaluate the efficacy of RVG29-SiO_2_-PEG-AuNR in the context of GBM, especially considering that GBM cells express functional nAChR (88). Sun et al. successfully integrated chelator-free ^64^Cu into Au nanomaterials of different sizes (10, 30, and 80 nm) and shapes (sphere, rod, and hexapod) for positron emission tomography (PET) imaging (46). Moreover, they tested RGD-peptide modified ^64^Cu- AuNR as GBM theranostic agents for PET-guided PTT after iv injection in U87 MG sc-grafted animals. Laser exposure (808 nm, 1 W/cm^2^, 10 min) was carried out 24 h post-injection and the spot size was adjusted to cover the tumor area. HT and tumor growth delay were achieved, even though the temperature mapping clearly shows temperature heterogeneity within the tumor upon laser exposure (ranging from 39.6 at bottom of the tumor to 60°C on the tumor surface; thermal camera) which might explain why tumors never disappeared completely. Unfortunately, no long-term survival studies were performed therefore it is unclear whether this partial PTT-mediated tumor ablation was sufficient to completely stop tumor growth.

Lu et al. used cyclic arginine-glycine-aspartic acid (RGD) grafted hollow gold nanospheres (cRGD-PEG-HAuNS) for photoacoustic (PA) tomography and PTT in an orthotopic GBM model (69). Their studies are carefully performed and present multiple controls. For example, to confirm the selective uptake of the particles in the tumor and the accuracy of PA imaging, cRGD-PEG-HAuNS were labeled with the positron emitter ^64^Cu and the tumor imaging was performed by CT/PET. Bioluminescence imaging was also used to visualize the tumor burden before and after treatment. NIR laser irradiation in mice 24 h-post injection with cRGD-PEG-HAuNS led to high tumor temperature increase (T_max_: 57.75°C; measured by magnetic resonance temperature imaging), leading to increased animal survival compared to the control groups. Despite this, all tumors eventually regrew leading to animal’s death, suggesting the presence of residual tumor cells after treatment. However, this theranostic system seems promising and could be combined to chemotherapy or standard of care treatment to increase its therapeutic efficiency. Day et al. also reported promising *in vivo* results on a sc U373 GBM model using AuNS and NIR laser irradiation (47). AuNS (150 nm, peak absorption at 800 nm) were administered systemically and they efficiently accumulated in the tumor within 24 h. Then, tumors were exposed to the laser beam (808 nm, 4 W/cm^2^, 3 min). AuNS-mediated PTT induced tumor regression and led to improved animal survival compared to the controls (tumor size measurement, bioluminescent imaging). Later, the same authors conjugated AuNS to Vascular endothelial growth factor (VEGF), to obtain an active targeting PTT carrier able to disrupt vasculature in orthotopic gliomas (70). *In vivo*, they demonstrated the efficacy of this system on a U373 GBM orthotopic model following systemic VEGF-AuNS administration and irradiation through a cranial window 24 h after treatment (808 nm, 6 W/cm^2^, 3 min). The set-up of a glass window (4–5-mm width/length) enabled laser light to be delivered to the tumor and to monitor tumor growth by vital microscopy in addition to bioluminescence. All treatment parameters were carefully selected (*e.g.* treatment dose, administration time, tumor size at treatment, laser beam diameter) to obtain the best exposure conditions. VEGF-AuNS efficiently bound to their target receptors on vascular endothelial cells and, upon laser exposure, were able to induce tumor vessels disruption reducing tumor growth while leaving the normal brain unharmed.

Nie et al. used plasmonic AuNSt conjugated with RGD peptide for PTT and PA imaging of tumor angiogenesis, tumor diagnosis, and treatment monitoring (48). *In vivo* studies on a xenograft sc U87 MG model showed high accumulation of the RGD-AuNSt at the tumor site following systemic treatment, allowing sensitive angiography. Six hours following systemic administration of RGD-AuNSt, continuous wave laser exposure (790 nm, 1 W/cm^2^, 10 min) induced a tumor temperature increase up to 45°C. Localized bleeding corresponding to the size of the laser spot was visible after laser irradiation, indicating local destruction of blood vessels at the exposure site. No temperature increases or PA angiography differences were reported in other parts of the body or in the control groups, and repeated administration doses significantly increased mice survival.

Yang et al. developed and precisely characterized a novel semiconducting-plasmonic nanovesicle formed by self-assembly of amphiphilic Au NP grafted with PEG and poly(perylene diimide) (PPDI-PEG-Au NP) (49). Collapsed absorbing PPDI and AuNP formed the vesicular shell, where a highly localized and strongly enhanced electromagnetic (EM) field was distributed between adjacent AuNP. This EM field enhanced the light absorption efficiency of PPDI, leading to increased PTT effect and PA signal compared to the mixture of single components (PDI NPs and Au vesicles). PA imaging was used to track the tumor accumulation of the vesicles and to provide information regarding tumor parameters (i.e., size, shape, extent of neovascularization), which is essential to adjust the laser spot size and power density of PTT to reach the best performance. Following iv administration in U87 MG-sc tumor bearing mice, a strong PA signal showed even distribution of the vesicles in the tumor up to 70 h post-injection, demonstrating that PPDI-PEG-AuNP vesicles can act as an excellent PA imaging probe for 3D reconstruction of the tumor. Moreover, NIR laser exposure of the tumor 30 h post-injection led to high temperature increase (T_average_: 50°C; T_max_: 80°C; IR camera) and excellent PTT effect with tumor growth suppression.

### Carbon-Based Nanomaterials

Akhavan et al. fabricated different shapes of rGO (nanoribbons, NR; nanomesh, NM) and functionalized them with amphiphilic PEG polymer chains, RGD peptide, and cyanine dies for active targeting, fluorescence imaging, and GBM PTT (50, 89). rGONM, which are graphene sheets with average lateral dimension of 61 nm and 0.9 nm thickness and containing 8 nm pores, showed to be very promising for this purpose as they possess strong NIR absorption at ultralow concentration (10 μg/ml) and ultralow laser power density (808 nm, 0.1 W/cm^2^, 7 min). The systemic administration of rGONM-PEG-Cy7-RGD in mice bearing sc U87 MG tumors followed by laser exposure resulted in efficient imaging, PTT and high therapeutic efficacy (100% long term survivors). Su et al. used porphyrin immobilized planar nano-rGO sheets conjugated with RGD (PNG-RGD) for targeted GBM PTT (51). The PTT efficacy of PNG-RGD was tested in a U87 MG sc tumor following its administration, showing that PNG can effectively reduce tumor growth compared to the controls. However, the author’s claim that particles can accumulate in the tumor in higher amounts than in the other organs was not properly demonstrated as the PNG-RGD administration was performed in high amounts and intratumorally (100 µl treatment for 6 mm diameter tumor). Yang et al. developed PEGylated nanographene oxide (PEG-NGO) loaded with anti-epidermal growth factor receptor (EGFR) monoclonal antibody cetuximab (C255) and epirubicin (EPI) for GBM (52). This triple treatment nanomedicine combines: C225 to target malignant glioma cells and inhibit their propagation; EPI chemotherapeutic drug delivery to damage cancer cells DNA; and PTT to induce local HT and kill residual tumor cells. EGFR expression was verified in several GBM cell lines (GL261, C6, U87 MG, and T98) by quantitative western blot and immunofluorescence to select a targeting cell model and the binding efficacy of C225 to EGFR on U87 MG cells was confirmed. *In vivo* therapeutic efficacy of triple-therapeutics C225-EPI-PEG-NGO was tested on a U87 MG sc model following systemic treatment administration and laser exposure at 3 days post-injection. The temperature in the tumor region increased up to 88°C at the tumor surface (thermal camera) after 120 s of laser irradiation in C225-EPI-PEG-NGO injected mice leading to tumor growth inhibition. Black scars remaining on the original tumor site healed within 7 days from treatment and tumors did not regrow over 50 days. Moreover, no body weight loss or signs of toxicity were observed demonstrating the potential of this system. Dong et al. developed a transferrin-conjugated PEGylated nanoscale graphene oxide drug delivery system loading DOX (TPGD) for combined chemotherapy and PTT in GBM (71). They showed successful cellular uptake in C6 and brain microvascular endothelial (BMVE) cells and strong transportability across the BBB in a BMVE *in vitro* model, which was further increased upon laser irradiation. The temperature measured in C6 tumor-bearing rats during laser exposure, 12 h post- TPGD injection, only increased from 34.15 to 38.10°C in the focal region (thermal texture mapping system). This might be due to the low distribution of TPGD in brain tumor tissue (which remains at nanogram level despite the active targeting) and to the slow variation of temperature due to the hard rat skull. Despite this, the authors showed that this PTT effect combined to the DOX release was enough to extend the survival time of rats bearing C6 tumors repeatedly treated by systemic TPGD and irradiation (808 nm, 2.5 W/cm^2^, 5 min).

Qian et al. developed multicolor highly crystalline carbon nanodots (HCCDs) (72). Their solid-state synthesis under high temperature was optimized to obtain a highly crystalline carbon nanocore and a hydrophilic surface. HCCDs have high water dispersity, good biocompatibility, exhibit tunable full color emissions, strong PA effect, and high photothermal performances. Their uniform small size (6–8 nm) allows penetration of the BBB gaps *via* passive targeting leading to high and prolonged accumulation in brain tumor tissues following systemic administration, useful both for tumor fluorescence and/or PA imaging and PTT treatment. *In vivo* antitumor efficacy studies on orthotopic U87 MG glioma-bearing mice demonstrated prolonged animal survival following treatment with HCCDs and NIR irradiation (72). The same group also developed PEGylated oxidized nanocrystalline mesoporous carbon particles linked to the Pep22 polypeptide (OMCN–PEG–Pep22) (73). OMCN–PEG–Pep22 were able to target the low-density lipoprotein receptor (LDLR), which is overexpressed in BBB endothelial cells and GBM cells, leading to efficient BBB-crossing and high tumor uptake. This system was loaded with doxycycline, and the combined chemotherapy and PTT effect was demonstrated upon systemic administration and multiple NIR laser exposure in orthotopic and sc glioma-bearing animals. This work clearly shows the complexity that comes along the development of effective PTT strategies for GBM. Indeed, the authors carefully selected the appropriate experimental conditions to be used to achieve safe and optimal temperature increase, measuring the scalp and glioma temperature with and without skull removal (using thermocouples to record intracranial temperature and a thermal imager) to select appropriate laser power density.

### Hybrid or Other Nanomaterials

As a way to increase the photothermal performance of rGO, Song et al. loaded DOX-containing rGO in ultrasmall AuNRs vesicles (rGO-AuNRVe-DOX) (53). rGO-AuNRVe-DOX were tested as PA contrast agents and dual chemotherapy and PTT agents in GBM. The plasmonic coupling of the conjugated small AuNRs in the vesicular shell combined with the interaction of the encapsulated rGO and plasmonic shell led to enhanced PTT effect and PA signal. Moreover, the encapsulation of rGO-DOX into AuNRVe avoided direct interaction of rGO-DOX with the physiological environment during the circulation and cellular internalization of DOX. A controlled and sequential release of DOX was triggered first by laser exposure (which disrupted the vesicle integrity) and later by the acid microenvironment (which released DOX from the rGO surface). The accumulation of rGO-AuNRVe-DOX in the tumor region after iv injection into U87 MG sc tumor-bearing mice was detected by a strong 3D PA signal, which showed the whole morphology and shape of the tumor. The PTT and combined chemo-PTT performance of rGO-AuNRVe-DOX was also tested on the same animal model, showing mild heating (45°C; thermal camera) but excellent antitumor efficacy at relatively low laser power density (808 nm, 0.25 W/cm^2^, 5 min). This temperature was high enough to trigger the disruption of the vesicle, leading to the release of DOX. At higher laser power densities (808 nm, 0.5 W/cm^2^, 5 min), the recorded tumor temperature was much higher (57°C) but led to the same result in term of long-term tumor eradication.

Another multifunctional nanocomposite formed of AuNR-coated Fe_3_O_4_ NS was developed by Hu et al. (54). pDNA-loaded AuNR- Fe_3_O_4_NS showed to be useful as contrast agent for multimodal imaging (X-ray CT, MRI, and PA) in C6 sc tumor-bearing mice. Combined gene therapy and PTT of this system were also confirmed on the same model following multiple it administration of pDNA-loaded AuNR- Fe_3_O_4_ NS and laser irradiation (808 nm, 2 W/cm^2^, 5 min) after the first treatment administration, leading to effective inhibition of C6 glioma. Lu et al. developed C225-functionalized core-shell Fe_3_O_4_@Au magnetic NPs (C225-Au-MNP) for active targeting and combined magnetic fluid and NIR HT (55). Selective uptake in cancer cells overexpressing the EGFR was confirmed *in vitro*. The therapeutic efficacy was demonstrated in a U251 sc model following peritumoral treatment followed by triple exposure of alternating magnetic field (f = 230 kHz, I=30 A; 30 min) and NIR laser (635 nm, 0.3 W/cm^2^, 30 min) with a time interval of 24 h. Wang et al. synthetized Iode-^125^ radiolabeled RGD-grafted PEG-Fe@Fe_3_O_4_ NPs (^125^I-RGD-PEG-MNPs) as multifunctional platform for MR/SPECT imaging guided GBM PTT (56). MR/SPECT imaging on a U87 MG sc GBM model confirmed the ^125^I-RGD-PEG-MNPs maximum tumor uptake at 6 h post-injection. PTT efficacy studies were performed at this time point by irradiating the whole tumor, which induced a tumor temperature increase of ∼8.2°C (IR thermal camera). Multiple PTT (three doses of treatment followed by laser irradiation) led to eradication of tumors 16 days post-treatment.

Hao et al. developed a dual chemotherapy and thermal therapy hybrid nanocomposite formed of docetaxel (DTX)-loaded poly (lactide-co-glycolide) (PLGA) NP with a discontinuous Au nanoshell (Au-PLGA-DTX NPs) (57).The tumor targeting peptide angiopep-2 (ANG) was also grafted on the nanosystem to confer it active targeting capability. The drug release from ANG-Au-PLGA-DTX NPs could be triggered by the NIR laser exposure, which led to destruction of the NP and accelerated DTX release. In a sc U87 MG model, the systemic administration of ANG-Au-PLGA-DTX NPs followed 4 h later by laser irradiation significantly reduced tumor growth thanks to localized HT (tumor temperature: 46.6°C, thermal camera). Moreover, the system showed potential as X-ray imaging agent showing its potential as theranostic tool.

Liu et al. designed cesium-based upconversion nanoparticles (UCNP) as a synergistic cancer theranostics nanoplatform for multimodal imaging (X-ray CT imaging and unique upconversion luminescence, UCL), chemotherapy, and PTT in GBM (58). The UCNP were grafted with RGD peptide for active targeting and loaded with chemotherapy drug alpha-tocopheryl succinate (α-TOS) and PTT coupling agent ICG. UCNP-PEG-ICG-TOS-RGD showed good tumor uptake in a sc U87 MG model 24- and 48 h post-iv administration and CT and UCL-imaging could be used to visualize the tumor 30 min post-injection. Moreover, a temperature increase up to 48.8°C was measured by thermal imaging IR camera. Repeated treatment showed increased antitumor efficacy for the UCNP-PEG-ICG-TOS-RGD + laser group compared to the controls (untreated, UCNP-PEG-ICG-RGD + laser, UCNP-TOS-RGD) in a small cohort of sc tumor-bearing animals. Tsai et al. developed hybrid nanoassemblies (ANG-IMNPs) for externally triggered targeted PTT GBM therapy. ANG-IMNPs are composed of oleic acid-coated UCNPs conjugated with PEG/ANG and loaded with photothermal agent IR-780 and photodynamic sensitizer 5,10,15,20-tetrakis(3-hydroxyphenyl) chlorin (mTHPC). ANG allows dual targeting effects on endothelial and glioma cells, increasing the drug delivery system uptake into the tumor. UCNPs can transfer energy to organic photosensitizers through resonance energy transfer upon 980 nm excitation, thus improving phototherapy. ANG-IMNPs were tested on ALTS1C1 murine astrocytoma cells *in vitro* and *in vivo*, showing enhanced cellular uptake, excellent accumulation at the tumor site and increased survival time of orthotopic tumor-bearing mice compared to the controls (e.g. 24 days *vs* 14 days of the non-targeted system) (74).

A responsive multifunctional organic/inorganic nanohybrids for multimodal guided-imaging (X-ray computed tomography CT, fluorescent and PA imaging) and triple-combination cancer treatment (PTT, gene therapy, and chemotherapy) was developed by Duan et al. (59, 60). This system is composed of AuNR, mesoporous silica (loading DOX), quantum dots, and two-armed ethanolamine-modified poly(glycidyl methacrylate) with cyclodextrin cores (exploitables as gene carriers *e.g.* antioncogene p53). Beside the potential of combining so many properties in only one nanocarrier, the heat generated by the NIR-laser exposure could disassemble the nanohybrid triggering DOX release. ASQ-DOX-PGEA2/p53 system was successfully tested for PTT/chemo/gene therapy *in vitro* and *in vivo* on a sc C6 model following repeated it treatment administration and NIR laser exposure. An IR camera was used to obtain the whole-body thermal images at different time points and a significant reduction of tumor volume and weight was observed at the end point, 8 days following the first treatment administration.

Tang et al. developed ^125^I radiolabeled self-assembling NPs formed of NIR dye croconaine, targeting peptide RGD and PEG (^125^I RGD-CR780-PEG) (61). This system could efficiently target U87 MG sc tumors *in vivo* as confirmed by NIR fluorescence and SPECT/CT imaging, while blood vessel distribution of the NPs in the tumor area was confirmed by PA imaging. The *in vivo* PTT effect was demonstrated following iv RGD-CR780-PEG administration and laser irradiation at low power density (808 nm, 0.5 W/cm^2^, 10 min) 6 h post injection. The tumor temperature increased up to 50°C (IR thermal camera) which led to dark-red skin at the tumor site followed by complete scarring and tumor eradication without regrowth in the next 40 days. Significant tumor-free survival times were obtained compared to all controls. Interestingly, the tumors in the passive-targeting group (Tyr-CR780-PEG) re-appeared with delayed growth further demonstrating the impact of highly selective and targeted tumor microvasculature destruction for long-term therapeutic efficacy. Jin et al. developed a melittin and ICG containing peptide nanofiber hydrogel as GBM PTT agent as well as PA contrast agent for temperature monitoring (62). This system is formed of a RADA16-I synthetic amphiphilic peptide that self-assembles into a hydrogel, containing melittin in its backbone and ICG in the matrix. The authors confirmed the PA properties of the hydrogel 3 h following its administration in a C6 glioma sc mouse model. The PTT efficacy was evaluated following its administration and laser exposure 24 h later (808 nm, 2 W/cm^2^, 8 min). This protocol led to increased tumor temperature in the treatment groups (53°C, IR thermal imaging camera) which generated tumor eschars that eventually healed, and significant tumor growth reduction. Wang et al. developed ultrasmall copper sulfide NPs loaded inside the cavity of ferritin nanocages (CuS–Fn NCs or ^64^CuS–Fn NCs) for multimodal imaging (PA, PET) and PTT (63). These bioinspired multifunctional CuS–Fn NCs showed good biocompatibility, strong NIR absorbance, high photothermal conversion efficiency and strong PA/PET contrast. *In vivo*, CuS–Fn NCs highly accumulated in U87 MG sc tumors following iv administration. Laser exposure 8 h post-injection led to temperature increase up to 65°C (thermal camera) which induced complete tumor eradication. Zha et al. grafted the NIR-absorbing nanomaterial (polypyrrole) on a ready-made photothermal ultrasound contrast agent *via* a one-step microemulsion method (64). By combining the properties of its two components, polypyrrole hollow microspheres (PPyHMs) could act as theranostic agents for both ultrasound imaging and PTT. In a U87 MG sc tumor model, it administration of PPyHMs followed by NIR laser exposure induced tumor temperature increase (70°C, IR thermal images) which led to significant tumor growth inhibition compared to the controls.

Zhu et al. developed holo-transferrin-indocyanine green (holo-Tf-ICG) nanoassemblies for targeted fluorescence and PA dual-modal imaging and GBM PTT (65). The targeting and imaging ability of holo-Tf-ICG was tested *in vivo* on two U87 MG models (orthotopic and sc) showing high fluorescent signal in the tumor region following systemic treatment administration, reaching a peak at 24 h post-injection. Moreover, the increased PA signals at 24 h after injection visually exposed the distribution of ICG within and outside the tumor microvessels. The PTT efficacy was demonstrated in a U87 MG sc tumor following iv administration followed by laser irradiation (808 nm, 0.8 W/cm^2^, 5 min) 24 post-injection. The temperature of holo-Tf-ICG NPs treated tumors increased above 55°C after 5 min of laser irradiation (IR thermal camera) leading to tumor growth suppression.

Guo et al. developed several generations of donor-acceptor conjugated polymeric nanoparticles (CPNP) with strong absorption in the second NIR window for precise PA imaging and PTT (66, 67). One of these NPs was decorated with the cRGD peptide to ensure active targeting of endothelial cells of the brain tumor vasculatures and GBM cells. cRGD-CPNP showed good photothermal conversion capability and photothermal stability after long or multiple cycles of laser exposure. Moreover, cRGD-CPNP show higher penetration efficiency through scalp and skull using 1064 nm laser instead of the most commonly used 808 nm (64 *vs* 75% light energy loss in nude mice, respectively). *In vitro*, the authors showed high tumor uptake of cRGD-CPNP in U87 MG cells, good biocompatibility and as well as PTT efficacy following 1,064 laser exposure. Moreover, extensive and well-conceived *in vivo* studies using a wide range of imaging techniques (MRI, bioluminescence, fluorescent and PA imaging) were performed to show that cRGD-CPNP followed by 1,064 nm laser irradiation is capable of effectively target and destroy brain tumors, without harming the healthy tissues. RGD-CPNP showed good biocompatibility, hemocompatibility, and IR thermal images confirmed high localized heating of 3 mm deep tumors following laser exposure 24 h post-injection (48.4 *vs* 39.7°C of control animals). This PTT effect inhibited tumor growth both in the U87 MG sc and orthotopic models, where treated animals showed improved survival compared to the controls (67).

Finally, Jia et al. recently developed biomimetic proteolipid nanoparticles (BLIPO-ICG) as a phototheranostic nanoplatform for brain tumor-specific imaging and therapy (75). BLIPO-ICG are formed of liposomes grafted to the imaging agent ICG incorporated with C6 glioma cell membrane proteins for crossing BBB and active targeting (*via* self-recognition to homologous cancer cells). They are biocompatible and biodegradable, possess high deformability and phototheranostic properties (active targeting, PTT-inducing properties, fluorescence imaging, and tumor margin detection). The tumor accumulation of BLIPO-ICG was evaluated in orthotopic GBM models at different tumor stages, to confirm the high homotypic targeting and immune escape properties of this system independently on the BBB disruption rate. Indeed, BLIPO-ICG accumulated in the tumor where they bound to the homotypic C6 glioma cells 12 h following systemic administration allowing tumor imaging as well as clear detection of the tumor margins for precise neurosurgery. Moreover, the authors demonstrated high PTT efficacy and tumor growth inhibition both in a C6 sc and orthotopic model (tumor temperature 57.2 and 47.9°C, respectively; thermal camera). In this last model, the scalp of the mice was removed for laser irradiation, but the skull was maintained and the laser beam spot was adjusted on the burr injection hole. Unfortunately, no survival analysis was performed on the orthotopic model as animals were euthanized 15 days after treatment but at this time point tumor size was significantly reduced in the treatment group compared to the controls.

## Discussion

As described in the previous sections, several studies have reported the use of innovative PTA NPs for GBM. The development of PTA NPs is challenged by the fact that they must possess PTA properties (optical properties, strong NIR absorption, large extinction coefficient, excellent photostability, good thermal conductivity, and effective generation of acoustic waves) as well as chemical composition and physicochemical properties (size, geometry, surface charge, hydrophobicity, biocompatibility, stability in biological fluids) adapted for systemic administration. They should bypass the BBB to reach the tumor site at therapeutic concentrations and show selective uptake in cancerous cells to avoid nonspecific heating of healthy cells (36). Moreover, they should also present good reproducibility and ease to scaling up, to increase their potential translation from bench to bedside (66). It is clearly visible from the number and complexity of the papers reporting NPs for PTT GBM (**Tables 1**–**3**) that bridging the *in vitro*-*in vivo* gap in this domain is extremely challenging. Indeed, multiple expertise (*e.g.* drug delivery, imaging, optics, pharmacology, cellular biology) are required to test such systems and the optimization of several individual steps is essential before combining them to demonstrate their PTT efficacy for GBM. It is interesting to observe how authors added certain features to the same system over time, to overcome the GBM challenges one by one [*e.g.* (50, 89); (47, 70); (66, 67)]. In general, authors start by describing the potential of their PTA NPs performing *in vitro* cellular uptake and PTT cytotoxicity studies (**Table 1**). Then, they demonstrate the proof-of-concept of the system *in vivo* using sc GBM models following it or iv administration and NIR laser radiation (**Table 2**). Finally, some groups manage to demonstrate PTT anticancer efficacy in orthotopic GBM models exposing the brain to laser exposure with or without scalp and skull (**Table 3**).

Considering the complexity of GBM, targeted therapies have been developed to bypass the BBB and actively target tumor cells to achieve maximum NPs tumor uptake. These strategies rely on grafting targeting ligands (*e.g.* antibodies, proteins, peptides) on the NPs surface to selectively recognize receptors overexpressed in a certain cells population (90, 91). Targeted drug delivery can specifically target brain capillary endothelial cells, tumor neovascular endothelial cells, and/or cancer cells (GBM or GSC cells) to bypass the BBB, the blood–brain tumor barrier or directly access the tumor, respectively. Some of the most common targeting moieties for GBM are Tf, FA, RGD, ANG, and IL-13 peptides (90). Other strategies can also be used to circumvent the BBB and enhance intracerebral NPs concentrations, such as the local delivery into the brain (*e.g.* by direct intracranial administration or convection-enhanced delivery) (92) or the use of focused ultrasounds combined with intravascular microbubbles (93). Recently, Yuan et al. also developed PTA NPs capable to increase BBB permeability. They tuned plasmon maximum peak of PEG- AuNSt to 800 nm to match their laser excitation system and to modulate AuNSt delivery into the brain *via* plasmonics-enhanced low-energy pulsed laser treatment (94). Focusing ultrafast pulsed laser (800 nm, 140 fs pulse width, 80 MHz repetition rate, 35 mW; 14 W/cm^2^) on brain tumors through a cranial window in mice preinjected with low dose of PEG-AuNSt (<1 pmol) induced a locally triggered microvascular permeabilization increasing brain accumulation and deep AuNSt permeation into tumor parenchyma (10–30 mm) with minimal off-target distribution. By tuning the laser parameters, it would be interesting to evaluate if PEG-AuNSt also show PTT effect in an orthotopic GBM model following systemic administration, BBB permeabilization and therapeutic PTT laser exposure. Moreover, it would be interesting to evaluate if other PTA NPs can have a double role of laser-inducing BBB permeabilization agents and PTT laser-induced anticancer effect.

Some authors have tried to combine PTT with imaging probes to obtain theranostic agents to better visualize NPs distribution or with other therapeutic strategies (*e.g.* chemotherapy, gene therapy) to increase the therapeutic outcome. The temperature changes are most often monitored by IR cameras. This technique is easily accessible but allows a superficial monitoring therefore it can be used for NP suspensions, *in vitro* cellular studies and sc tumors but is not suitable for intracranial tumors (*e.g.* orthotopic GBM tumors). To monitor tumor temperatures within the brain nanothermometers or advanced imaging techniques are needed. For example, Schwartz et al. reported a pilot study performed on an orthotopic canine model of brain tumor (canine transmissible venereal tumor) using AuNS and PTT (95). Intracranial tumors were thermally ablated by percutaneous, optical fiber–delivered, NIR radiation (808 nm, 3.5 W, 3 min) 24 h following AuNS iv administration. The temperature-dependent proton resonance frequency shift was used to record temperature changes and processed to display temperature maps using a thermal mapping system: the temperature at the tumor site was selectively elevated up to 65.8°C following AuNS PTT treatment, showing highly specific brain tumor ablation. Even though this study was not performed in a GBM model, and the scalp/skull were avoided by direct irradiation of the tumor site—which requires invasive surgery—the authors were able to monitor temperature variations in the brain in real time by magnetic resonance thermal imaging bypassing one of the BBB PTT challenges. Another emerging imaging approach that could be exploited is PA imaging. In this review we highlighted how PA imaging enables multiscale high spatial and deep resolution imaging of tissue and biological structures (49, 69). However, this technique can also be used to track the temperature-induced changes in PA signal amplitude and provide a control of temperature increase following laser exposure (96, 97).

To conclude, PTT might be a promising strategy also for GBM but its success is limited by several challenges. In this review, we identified three of them: *i)* the accumulation of PTA NPs into the tumor is restricted by the BBB; *ii)* the monitoring of the temperature increase inside the brain is not a simple task but is essential to obtain safe and efficient PTT treatments; *iii)* localized HT must be sufficient to achieve cancer cells death even if the tumor localization is deep in the brain. How systems were developed and tested to overcome these barriers was critically discussed to have a clear vision of the advancements in this field. While it is unlikely to expect that any of the reported systems will lead to complete eradication of GBM—because of the high number of infiltrative cells invading the peritumoral region and non-cancerous subpopulation resident within the tumor mass—we hope that selective targeting and PTT could lead to partial ablation of the tumor. The partial tumor destruction and the immunomodulation triggered by the PTT, combined with chemo-, radio-, or gene-therapies could potentially lead to increased therapeutic outcomes.

## Author Contributions

CB conceived, wrote, and edited the manuscript. All authors contributed to the article and approved the submitted version.

## Funding

The authors acknowledge the financial contribution from ITMO cancer AVIESAN (National Alliance for the Life Sciences & Health) within the framework of the Cancer Plan (GRAVITY Project).

## Conflict of Interest

The authors declare that the research was conducted in the absence of any commercial or financial relationships that could be construed as a potential conflict of interest.
